# Supplementary material for: Survival status and predictors of early neonatal mortality among neonates admitted to neonatal intensive care units in Addis Ababa Public Hospitals, Ethiopia: A retrospective cohort study
Source: PLoS One. 2025 May 28;20(5):e0323600. doi: 10.1371/journal.pone.0323600 (PMC12118970; doi:10.1371/journal.pone.0323600)
Supplement: Attachment — Submitted filename: A rebuttal letter for each comments raised by an a.pdf [file pone.0323600.s003.pdf]

A rebuttal letter for each comments raised by an academic editor, PLOSE ONE

Dear, academic editors of the PLOSE ONE journal, This is Ashenafi Seifu Gesso a corresponding author for the manuscript entitled “Survival status and predictors of early neonatal mortality among neonates admitted to neonatal intensive care units in Addis Ababa public hospitals, Ethiopia 2023: A retrospective cohort study”. It’s a great honor to work with this journal and I have publications previously as well. This journal is one of the most respected and reputable journals we are considering to publish our scholarly articles. As such, I am recommending friends, and colleagues to join working with PLOSE ONE journal. Below points are point by point responses for editors comments.

1. When submitting your revision, we need you to address these additional requirements.

Response- I was not strictly followed Plose one authors guidelines at the prior submission. Now, in the revised submission I did it as per the journal standards.

2. We note that your Data Availability Statement is currently as follows: "All relevant data are within the manuscript and its Supporting Information files"

Response= In the revised version I stated it as “The datasets used and/or analyzed during the current study are available from the corresponding author upon reasonable request”.

3. Your ethics statement should only appear in the Methods section of your manuscript.

Response=Yes, it was stated almost twice in the prior submission, but corrected in the revised submission.

4. Please include captions for your Supporting Information files at the end of your manuscript, and update any in-text citations to match accordingly

Response= The prior submission provided supporting information separately. The revised submission added captions for supporting information at the end of the manuscript file.

Additional comments

Please, revise the PLOS ONE authors' guidelines and re-submit your manuscript. For instance; your manuscript:

-Has no line-numbers

-Not double spaced

-You did not follow the PLOS ONE guidelines to prepare the Figures and Tables

-Has many typographic and editorial errors

Response

The former submission had no line-numbers, revised one has continuous line numbers

The prior submission was 1.5 line spaced, but the revised one is double spaced

The former submission did not followed captions for tables and figures, but the revised one was inline with PLOS ONE guidelines to prepare the Figures and Tables

The first submission had some grammatical and punctuation errors, but the revised one solved those typographic and editorial errors.
