# Supplementary material for: Survival status and predictors of early neonatal mortality among neonates admitted to neonatal intensive care units in Addis Ababa Public Hospitals, Ethiopia: A retrospective cohort study
Source: PLoS One. 2025 May 28;20(5):e0323600. doi: 10.1371/journal.pone.0323600 (PMC12118970; doi:10.1371/journal.pone.0323600)
Supplement: Attachment — Submitted filename: Point by point response to reviewers.pdf [file pone.0323600.s004.pdf]

Point by point response to the editors and reviewers comments

1. If the authors have adequately addressed your comments raised in a previous round of review and you feel that this manuscript is now acceptable for publication.

Response: Yes we appropriately revised as per the comments given in the subsequent reviews .

2. Is the manuscript technically sound, and do the data support the conclusions?

Response: Yes, absolutely the conclusions were drawn from the results presented.

3. Has the statistical analysis been performed appropriately and rigorously?

Response: Yes, we performed survival analysis to see when will a neonate less than or equal to 7 days old would die after admission to neonatal intensive care units.

4. Have the authors made all data underlying the findings in their manuscript fully available?

Response: Yes, we declared it in detail and we can provide every data sets used for this analysis without restriction.

5. Is the manuscript presented in an intelligible fashion and written in standard English?

Response: Yes, we made this manuscript well articulated and informative through our publication experience and PLOS ONE standards.

6. Review Comments to the Author

Please use the space provided to explain your answers to the questions above. You may also include additional comments for the author, including concerns about dual publication, research ethics, or publication ethics. (Please upload your review as an attachment if it exceeds 20,000 characters)

Reviewer #1: All my comments and suggestion given in the first version, addressed in this version, no additional comments, concerns and suggestion on this version.

Reviewer #2: Dear editor I would like to thank you for inviting me to review this paper done by Gesso et. al. The paper tried to investigate incidence and predictors of mortality of neonates in neonatal ICU in Addis Ababa, Ethiopia, using a retrospective chart review. This manuscript brings unaddressed challenge of public health. However, the manuscript lacks fundamental point to merit publication.
Major issues
1. Lack of novelty:

Response: We see the concerns over the novelty, but the previous study was simple cross sectional study with incidence and associated factors for NICU admitted

neonates. The current study(ours) was survival study that looks when will the early neonate (from first day to day seven of life) die after admission to the NICU. Ou study was different in population, in methods, analysis and finding as well.

2. Study design: Almost all previously conducted studies used retrospective cohort study (chart review) recommended to further conducting prospective studies in order to better estimate incidence and identify predictors of neonatal mortality in the NICU. However, this recent manuscript used retrospective chart review which makes the manuscript less attractive.

Response: The current study (our was also retrospective), we understand and we stated the limitations of retrospective study in the methods section of the manuscript. But the objective was to pick the predictors of early neonatal mortality (≤7 days of life) not for all neonates admitted to the neonatal intensive care units.

3. Length of follow-up in NICU: The authors described neonates stayed in NICU a minimum of 1 day to a maximum of 7 days. This seems unrealistic since neonates, especially on phototherapy, sepsis, and neonatal enterocolitis, might stay more than 7 days. This raises a question on data collection.

Response: With regard to this concern, we put early neonatal mortality in operational definition in the manuscript. Accordingly, if a neonate stays for more than seven days we declared improved or stayed beyond 7th days of life, if not an event was recorded as died.

4. Result and Statistics: A poor presentation of results. It is not relevant to provide Log-rank test results if the authors run Cox regression. Not relevant to upload Kaplan-Meier graphs of categorical variables as a supplementary file. The authors did not assess variability across clinical settings using a statistical approach (This might lead to analysis with shared frailty model).

Response: We understand the concern from the reviewer, but we added Kaplan-Meier graphs to increase clarity for the readers as long as this would not alter the cox-regression findings.
